# Efficient Binary Weight Convolutional Network Accelerator for Speech Recognition

**DOI:** 10.3390/s23031530

**Published:** 2023-01-30

**Authors:** Lunyi Guo, Shining Mu, Yijie Deng, Chaofan Shi, Bo Yan, Zhuoling Xiao

**Affiliations:** School of Information and Communication Engineering, University of Electronic Science and Technology of China, Chengdu 611731, China

**Keywords:** binary weights, speech recognize, hardware accelerator, ZYNQ, multichannel shared computation

## Abstract

Speech recognition has progressed tremendously in the area of artificial intelligence (AI). However, the performance of the real-time offline Chinese speech recognition neural network accelerator for edge AI needs to be improved. This paper proposes a configurable convolutional neural network accelerator based on a lightweight speech recognition model, which can dramatically reduce hardware resource consumption while guaranteeing an acceptable error rate. For convolutional layers, the weights are binarized to reduce the number of model parameters and improve computational and storage efficiency. A multichannel shared computation (MCSC) architecture is proposed to maximize the reuse of weight and feature map data. The binary weight-sharing processing engine (PE) is designed to avoid limiting the number of multipliers. A custom instruction set is established according to the variable length of voice input to configure parameters for adapting to different network structures. Finally, the ping-pong storage method is used when the feature map is an input. We implemented this accelerator on Xilinx ZYNQ XC7Z035 under the working frequency of 150 MHz. The processing time for 2.24 s and 8 s of speech was 69.8 ms and 189.51 ms, respectively, and the convolution performance reached 35.66 GOPS/W. Compared with other computing platforms, accelerators perform better in terms of energy efficiency, power consumption and hardware resource consumption.

## 1. Introduction

Convolutional neural networks (CNN) [1] are the basis of biological vision [2,3], natural language processing (NLP) [4], and LeNet-5 [5]. VGG16 [6] used convolution kernels to build a very deep network model structure, proving that increasing the network depth can affect the final network performance to a certain extent. As an increasing number of complex neural network model structures have been proposed, the deployment of neural networks on edge devices has presented extraordinary challenges owing to their sizeable computational load and frequent memory access. High-performance computing devices, such as graphics processing units (GPUs), are generally used to train neural networks. However, GPUs are no longer an ideal computing platform for deploying neural networks, owing to their high-power consumption. In recent years, field-programmable gate arrays (FPGA) have attracted increasing attention from academia and industry concerning CNN accelerators [7,8] owing to the characteristics of many computing units and memory blocks.

The limited storage and computing resources of FPGA and the innovation of network models have challenged the generality and energy efficiency of neural networks. Many current neural network accelerators [9,10] improve speed and performance by incorporating lightweight network models and new hardware architectures. The Roofline model [11] can improve the overall system throughput by increasing the data transmission bandwidth and number of unit memory access calculations under different constraints. Ma et al. studied loop unrolling, tiling, and transformation in convolution operations [12] using the degree of data consumption and the parallelism between convolution kernels to improve the throughput of the accelerator. A neural network accelerator applies a reconfigurable design [13] to adapt to different network structure sizes. OPU [14] uses ping-pong storage for all feature maps, weights, and instructions to improve computational efficiency, but its excessive resource consumption leads to high system power consumption. Because of the sparsity of the neural network, skipping the calculation of zero values using the sparsity of the weight or feature map can improve computational efficiency [15], and the matrix size can be compressed using weight coding and nonzero bitmaps. The amount of computation also introduces storage space into the encoding matrix while increasing the complexity of computational control. Huang et al. [16] designed an accelerator architecture for multiengine pipeline computing and set dedicated parameters for each layer of the network, but this approach lacks generality.

Most of the existing binary neural network accelerators [17,18] have been chosen to be implemented on a 10-classification task dataset such as MINST or CIFAR-10. Such datasets have a low number of classifications and model parameters, and it is easier to achieve higher accuracy by binarizing the original full-precision neural network model. It is suitable for embedded devices with limited power and resources. However, more complex neural network models cannot be implemented on them.

In addition, some researchers [19,20,21] have also implemented binary neural network accelerators on datasets with 1000 classification tasks, such as ImageNet, but their energy efficiency needs to be improved.

As observed, the aforementioned research optimizes storage efficiency, versatility, parallelism, and quantization compression through various methods. Based on previous research, this study first establishes a neural network model for speech recognition tasks of classifying 1424 phonemes and then binarizes the model convolution weights to reduce the number of model parameters. Finally, a convolutional neural network accelerator based on ZYNQ is developed using an algorithm-hardware co-design. Energy-efficient and reconfigurable designs were achieved using various optimisation strategies. The main contributions of this study are summarised as follows.

- A neural network model for a Chinese speech recognition task was designed. The optimisation method of the model includes improving VGG-16 to reduce its fully-connected layer to one layer and increase the number of convolutional layers. The convolutional layer weights are binarized and then trained by the transfer learning method. The binary weighting design speeds up the network computation and dramatically reduces computational complexity and memory consumption.
- Taking full advantage of the parallelism of FPGA, a new multichannel shared computation (MCSC) architecture is proposed, which maps all network layers in a typical convolutional computing engine. In this structure, the accelerator can realise 576 multiplications in one cycle, and the ping-pong storage of the input feature map data reduces the idle time of the computing array and improves throughput.
- For the binary weight of the convolution layer, a sparse convolution calculation unit without a DSP was designed to realise binary multiplication with only seven Look-Up Tables (LUT). This design significantly reduces resource consumption.
- The accelerator customises control instructions between the 11 programmable logics and the processing system. Through the configuration of the instruction parameters, processing different-sized networks and cooperatively operating each module can be realised to achieve a reconfigurable design.
- According to the hardware architecture proposed in this study, the accelerator was implemented on a Xilinx ZYNQ XC7Z035. Under the operating frequency of 150 MHz, the delay for 2.24 s of speech was 69.8 ms, and the delay for 8 s was 189.51 ms. The convolution energy efficiency ratio reached 35.66 GOPS/W when the convolution layer used 0 DSP resources. State-of-the-art results have been achieved in FPGA-accelerated speech recognition research.

## 2. Related Work

### 2.1. Speech Recognition Neural Network

**Figure 1:**
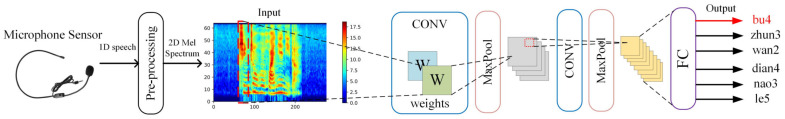
A typical Chinese speech recognition convolutional neural network where [bu4, zhun3, wan2, dian4, nao3, le5] is a Chinese Phoneme sequence, which in English means [stop playing computer].

The field of speech recognition based on neural networks has entered the end-to-end era in which the problem of inconsistent input and output lengths in speech recognition models is primarily solved by end-to-end technology. The end-to-end techniques mainly include connected temporal classification (CTC) and attention mechanisms, and the CTC-based speech recognition model adopts repeated labels and blank labels to identify blank frames for solving the sequence alignment problem [22]. Phoneme error rate (PER) in Chinese speech recognition is a standard evaluation indicator. The preprocessing method converts one-dimensional (1D) speech signals into two-dimensional (2D) Mel spectrum, which is the basis for speech signals to realise convolution calculations, such as images. Figure 1 shows the structure of a typical Chinese speech recognition neural network model. The microphone sensor receives the 1D speech signal and then converts it into a 2D Mel spectrum through a preprocessing module. The speech Mel spectrum was input to the neural network, and the Chinese phoneme sequence was the output. The fusion network structure designed by RRAINet [23] achieved a PER of 15.45% on the Chinese standard dataset, ST CMD [24]. The MCNN [25] proposed that multipath convolution achieves a PER of 22.97% on the ST CMD dataset. The end-to-end network structure composed of the VGG + CTC [26] combined with CNN and CTC loss functions has achieved 23.86% of PER on the ST CMD data set. CNN has achieved excellent results in speech recognition tasks, and most of the current recognition and classification tasks choose CNN to verify the performance of the accelerator.

### 2.2. Binary Neural Network

The BinaryConnect [27] method proposed by Matthieu et al. was the first to summarise the complete training process of a binary neural network. This paper proposes the use of 1-bit binary weights instead of 32-bit floating-point precision weights in the forward and backward propagation of a CNN. Hardware computing can simplify multiplication operations into accumulation operations and reduce storage space. Matthieu et al. [28] proposed a binarised neural network (BNN) to binarise the activation value. The complex convolution calculation is simplified to an XNOR operation and popcount operation suitable for hardware operation, reducing the hardware time complexity by 60%. Rastegari et al. [29] proposed that XNOR-Net implements binary values for both weights and activation parameters, which improves the inference speed by 58 times and saves memory 32 times on hardware. An accurate binary convolutional network [30] fits floating-point values by linearly combining multiple binary weights and activation values to reduce information loss. The accuracies of Top1 and Top5 obtained by this method on the ImageNet dataset were only 5% lower than the accuracy of the ResNet-18 original model. The proposed BDNN [31] for speech recognition tasks has a 5% increase in error rate compared to full-precision networks. ReActNet [32] used a new network model that combined residual connection and stitching methods, improved the traditional sign and PReLU functions, and achieved a Top-1 accuracy of 71.4% on ImageNet.

The binary neural network uses quantised weight values in both forward and backward propagation but maintains floating-point weights for updating during training. The training method using the binary neural network has become increasingly mature, and the gap between the inference accuracy and the floating-point precision network has gradually narrowed, implying that the binary neural network has achieved a specific application value. In this study, the weights were constrained to 1 and 0 for the task of Chinese speech recognition based on convolutional neural networks.

### 2.3. Hardware Accelerator for BNN

Fixed-point high-precision neural network accelerators have achieved significant computing energy efficiency. However, the core computing of high-precision neural networks is still dominated by high-cost and high-power multiplication operations and requires frequent access to off-chip storage. The binary neural network has low computational complexity and low storage requirements, which are very suitable for multiplier-free operations and reduces a large number of off-chip memory accesses. Therefore, the hardware acceleration of binary neural networks has been extensively studied.

The FPGA-based binary neural network hardware accelerator is represented by the FINN [33] architecture proposed by the Xilinx Research Lab. FINN is a fast and flexible heterogeneous dataflow neural network architecture. Through a series of architectural optimisations, the throughput on the MNIST and CIFAR-10 datasets reached nine TOPS and 2.5 TOPS, respectively, and the performances were 1032 GOPS/W and 685 GOPS/W. Fu et al. proposed a fast and efficient binary neural network inference architecture [34]. By reusing previous calculation results, many data buffer accesses and calculation cycles can be skipped, reducing the calculation amount by 80% and 40% of the cache access volume, respectively, and the throughput achieved on the FPGA reaches 975 GOPS. The resource efficiency is 1.9 times higher than that of advanced designs of the same type and 10 times higher than that of 16-bit accelerators. BinaryEye [35] is an FPGA-based streaming media camera system that uses a binary neural network to classify regions of interest within a frame, achieving massive data reduction with a throughput rate of 20,000 frames per second (FPS). The binary neural network accelerator built by Qiao et al. [36] can fuse batch normalisation layers and reduce the computational resources and memory requirements by 84.2% and 96.4%, respectively.

The research results of many binary neural network hardware accelerations show that binary neural network accelerators have more significant advantages than high-precision fixed-point accelerators in terms of throughput and hardware resources. However, FPGA-based accelerators mostly use data routing architectures, and there is room for improvement in terms of computational energy and resource efficiencies. In addition, owing to the application of binary quantisation, the accuracy of the model is not as high as that of a high-precision network. Furthermore, it is a new method to optimise the performance of a binary neural network with a full-precision network through transfer learning. At the same time, the energy efficiency of the binary neural network accelerator for complex data sets with multiple classifications must be further improved. The binary neural network has achieved excellent image classification and target tracking results. For the Chinese speech recognition task, this study designed an energy-efficient binary neural network hardware architecture and tested and validated the hardware architecture based on ZYNQ.

## 3. Binary Speech Recognition Network Architecture

### 3.1. Network Structure

In general, a CNN contains multiple convolutional, normalisation, activation, and pooling layers, each having different roles in the model. Feature extraction from input data using convolutional layers is an abstract representation of the source data relevance. The input to the convolution calculation is the feature map Fin(H,W,N), which is the data of N 2D matrices of length and width H and W. The input feature map is Finp(H+2,W+2,N) after padding. The parameters of the convolution filter are ω(Q,P,N,K), where Q and P are the length and width of the convolution kernels. In this paper, 3×3 is used as the size of the convolution kernels, N is the number of input convolution kernel filters, and K is the number of output convolution kernel filters. The output of the convolution calculation is the feature map Fout(H,W,K), which is the data of K 2D matrices of length and width H and W. The bias term is Bias(K). The kth feature output Fout(i,j,k) can be described as follows:

(1) $$Fout(i,j,k)=Bias(k)+\sum_{n=1}^{N}\sum_{q=1}^{3}\sum_{p=1}^{3}Finp(i+q-1,j+p-1,n)*\omega (q,p,n,k)$$

where ∗ is the convolution operation. The essence of the convolution calculation is multiplying and adding 2D filter sliding windows and summing them vertically, the result of which is the local feature information matrix extracted from the original matrix.

In this study, an acoustic model was constructed using VGG-16 combined with CTC. Because CNNs have excellent classification characteristics, they can fuse and extract the deep feature information of the speech Mel spectrum feature map. Simultaneously, combined with the characteristics of the CTC algorithm, CNN-CTC is suitable for the field of speech recognition. The parallelism of the fully-connected layer is less significant than that of the convolutional layer. The fully-connected layer of the model has only one layer to improve hardware throughput while ensuring the model’s accuracy.

CNN models generally perform floating-point calculations when trained or inferred using the software. However, floating-point calculations on FPGA require many resources; therefore, the model needs to be quantised to reduce the resources consumed by FPGA computing and storage. Convolutional neural networks have strong robustness, and the resource overhead can be reduced by reducing the bit width while ensuring accuracy. The classic binary neural network quantizes all weights and activation functions to 1-bit. Although this method can significantly reduce the number of parameters, it cannot guarantee the accuracy of the network effectively. Therefore, we designed BWN to limit the weight of the convolutional layer to 1-bit to avoid the need for DSP multiplication in the hardware and to reduce resource overhead. In BWN, the convolution weight is limited to 1-bit, according to Equation (2):

(2) $$w_{b}=\left\{\begin{array}{ll} +1, & \mathrm{if}\;w_{fp}\geq 0\\ -1, & \mathrm{if}\;w_{fp}<0 \end{array}\right.,$$

where $w_{fp}$ represents the original full-precision weights of the convolutional layer, and the network training generally includes three stages: forward propagation, backpropagation, and parameter update. In BWN, the binarised weights $w_{b}$ are used in both the forward and backpropagation stages, but the full-precision weights $w_{fp}$ are used in the parameter updates. We use the transfer learning method to retrain the binary weight neural network with the weights of the trained full-precision model. To ensure a balance between parameter quantity and accuracy, this study constrains the weight of all convolutional layers to 1-bit. However, the last fully-connected layer is quantised to 8 bits. The comparison results of the model structure and related parameters of the full-precision network and binary weight network are shown in Table 1. After the binarisation of all convolutional layers, the number of parameters is only 3.63% of the full-precision parameters. The number of multiplications and additions of the entire network is 13.25 G. However, the total number of parameters is only 11.81 M, of which the parameters of all convolutional layers are only 0.68 M.

As presented in Table 1, the acoustic model takes the complete sentence of a 16-KHz sampling rate speech as input, called a frame. The initial width and height of the network are H=800 and W=64, respectively, and the number of convolution channels is N=1. The feature dimension and frequency information are extracted in the subsequent 2D convolution calculation. The specific implementation steps of the acoustic model follow.

The first part of the model is a convolutional layer network, which reduces the size of the time and frequency dimensions and increases the number of convolution channels through convolution and pooling operations, which perform the primary feature extraction function. The output shape of the last layer is (100, 8, 256). The second part of the model is the Reshape layer, which converts the 3D data into a 2D output shape (100, 2048). The third part of the model is a fully-connected layer that acts as a classifier to obtain the output shape (100, 1424), which maps the learned distributed feature representation to the sample label space. The fourth part of the model is Softmax, which classifies the 1424 pinyin phonemes according to their probability magnitude. The input to the Softmax layer is Fins(H,S), and the output is Fouts(H,S). The output of the hth probability distribution Fouts(h,s) can be described by Equation (3).

(3) $$Fouts(h,s)=\exp(Fins(h,s))/\sum_{s=1}^{1424}\exp(Fins(h,s))$$

The Softmax function’s output value for the classification can be converted into a probability distribution in the range (0, 1), and the sum is 1. The result of the pinyin phoneme can be obtained depending on the position of the maximum value. Finally, the probability distribution is fed into the CTC to calculate the loss for backpropagation. Next, we compare the performance of weights and activations under quantisation for different bit widths.

### 3.2. Accuracy of Network

The dataset used in this work is the ST CMD released by an AI company Surfing Technology, which contains more than 100,000 speech files and approximately 100 h of speech data. The data content is mainly based on the usual online chat and intelligent speech control sentences. There were 855 speakers, including half male voice and half female voice, suitable for various scenarios. We experimented with various quantisations with different bit widths for the network weight and activation function. The model PER comparison is presented in Table 2, where W is the weight bit width, A is the bit width output by the activation function, and PER is the evaluation index of the speech model. The lower the PER, the better the network performance.

In Table 2, we can see that the difference in PER between customized VGG-16 at the data precision of Float 32-bit and 1W/Float 32-bit is insignificant. However, the data precision of 1W/Float 32-bit increases the PER by only 0.27% with an 18.03 M reduction in convolution parameters. 16-bit and 8-bit quantization of multibit-weighted network activation functions have little accuracy loss. For the binary weight network, the PER increases by 1.77% when the activation function is quantized to 16 bits, but the PER is not very different from that of the activation function 8-bit quantization. In addition, we found that when the activation function is fixed to 4-bits in the experiment, the recognition result cannot be predicted, and the data bit width in the hardware is generally 2^n^. Hence, the accelerator selects 1W/8A to quantify the neural network and conduct a performance comparison in the subsequent hardware implementation. LUT, Flip Flop (FF), and DSP are essential resources in FPGA. ZYNQ 7035 implements common 8W/8A multiplication in vivado2018.2 and consumes 72LUT or 1DSP of hardware resources. However, our designed 1W/8A multiplication consumes only 7LUT, which greatly reduces hardware resources.

## 4. Accelerator Architecture

In this section, the accelerator’s overall architecture and data transmission method are introduced, and the convolution calculation structure based on the sparse feature map data and binary weights is described. Subsequently, the MCSC architecture is proposed. Finally, a configurable instruction set is established to realise the reconfigurable design.

### 4.1. System Architecture of Accelerator

**Figure 2:**
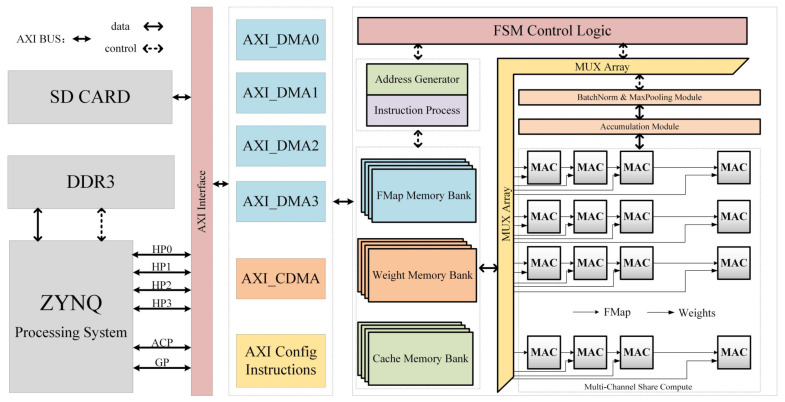
The overall architecture of SoC.

As shown in Figure 2, the entire neural network accelerator architecture primarily constitutes a processing system (PS), direct memory access (DMA), double data rate (DDR), controller, data storage block random access memory (BRAM), and a computing module. Red and black arrows represent the control and data lines, respectively. Among them, the PS distributes reconfigurable parameters, transmits feature maps and weight data through DMA based on the advanced extensible interface (AXI), and displays the final results. The accelerator mainly performs hardware acceleration of convolution, full connectivity, and Softmax. Each module of the accelerator is controlled by a finite state machine (FSM), which enables variable-length speech input and reconfigurable designs through different parameter configurations. In the core convolution calculation, we designed a binary processing engine (PE) that does not require a DSP. Finally, the entire neural network hardware accelerator was implemented in Verilog.

### 4.2. Data Transfer Architecture

Owing to the large scale of our speech recognition CNN model, the on-chip BRAM cannot store the weights and feature maps of all the intermediate layers. Therefore, when designing a CNN acceleration system, it is necessary to use the external DDR and on-chip BRAM for large-scale data exchange. The design and construction of the data transmission system affect the size of the data transmission bandwidth, which is one of the determinants of the acceleration effect of the CNN. In the ZYNQ platform, the AXI_GP interface is generally a low-speed communication; therefore, the AXI_GP interface is used for the control command interaction and parameter configuration of the accelerator. In contrast, the AXI_HP and AXI_ACP interfaces are high-speed and low-latency interfaces; therefore, four AXI_HP interfaces and AXI_ACP interfaces were used in this study for bulk weights and feature map data transfer. For high-speed memory access to large data batches, it is generally necessary to rely on DMA to directly control the bus to realise the memory transfer of large data batches to reduce the CPU load. In the ZYNQ platform, there are two commonly used DMAs: AXI_DMA and AXI_CDMA. The resource and performance comparisons between the different transmission methods are presented in Table 3.

**Figure 3:**
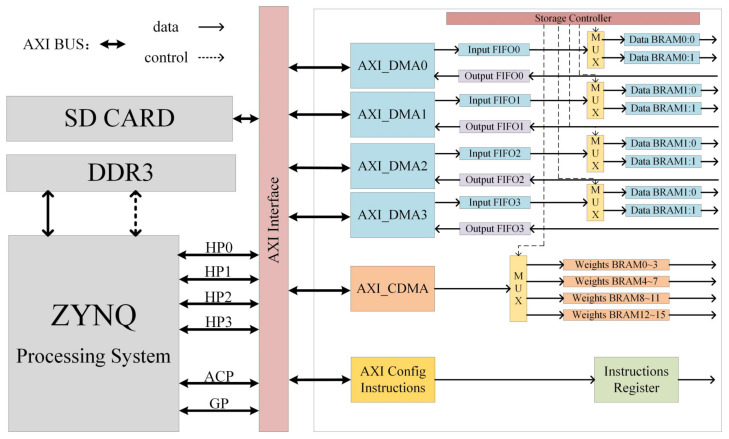
Data transfer architecture.

Based on the above characteristics of the ZYNQ platform, we built the CNN accelerator data transmission system, as shown in Figure 3, including four feature map transmission channels and one weight transmission channel.

For the feature map transmission channel, the four AXI_DMAs correspond to the four AXI_HP interfaces. Each feature map channel includes a Multiplexer (MUX), two feature map–ping-pong storage BRAMs, and an output feature map storage FIFO. For the weight transmission channel, AXI_CDMA corresponding to the AXI_ACP interface is used to map and transmit the weight source address to the destination address, including 16 weight storage BRAMs. In the data transmission system design, both the convolution layer and the fully-connected layer as well as the input and the output storage resources are multiplexed, which not only forms the basis of the MCSC architecture but also facilitates saving hardware resources.

### 4.3. Binary Convolution Compute Design

**Figure 4:**
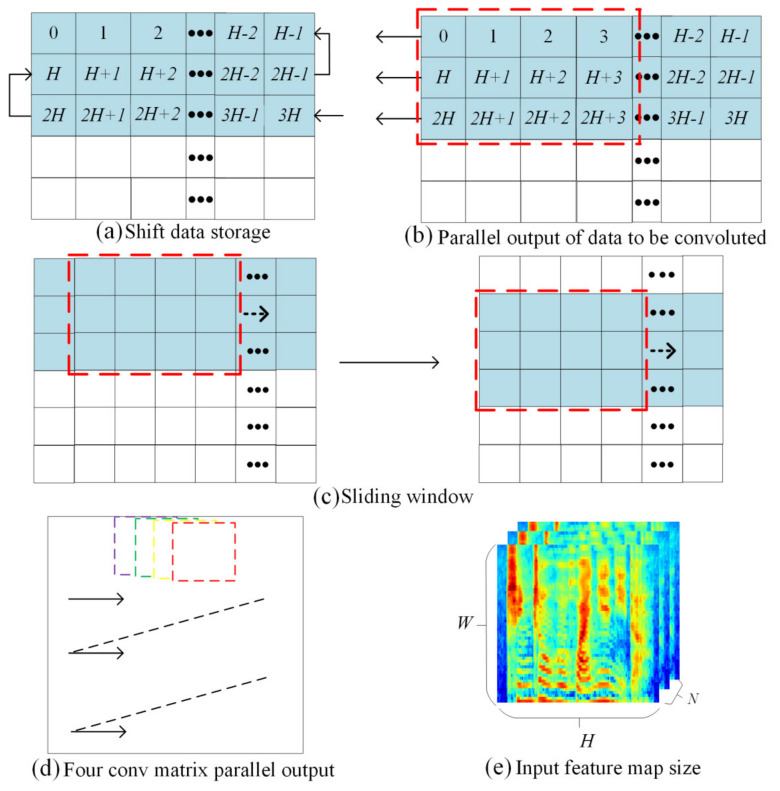
Convolution four-matrix parallel fetch.

The convolution kernel size in this design is 3×3, and the stride was 1. The convolution operation slides the convolution kernel on the input feature map and the convolution operation is performed on different local data. To update the feature map data that require convolution in real time, we designed a pipelined shift sliding window module, as shown in Figure 4. The shift sliding window module uses three shift RAMs to form a serial-in and parallel-out first input-first output (FIFO) to store and update the 3-line data of the input feature map, where W, H, and N are the numbers of rows, columns, and input channels of the input feature map, respectively. As shown in Figure 4a, the input feature map first stores all of the first three columns of data in the three shift RAMs. The fixed-point bit width of the feature map is 8 bits, and the bus data bit width is 32 bits. Therefore, as shown in Figure 4b, the data are output with a size of 3×4 in length and width. When all three columns of the feature map data are outputted, as shown in Figure 4c, the next column of the feature map data are input into the shift RAM, and the convolution matrix continues to slide the output data. To match the transmission bandwidth with the calculation bandwidth, this study reuses the overlapping sliding window data with the four convolution matrices shown in Figure 4d to slide in parallel and calculate simultaneously.

**Figure 5:**
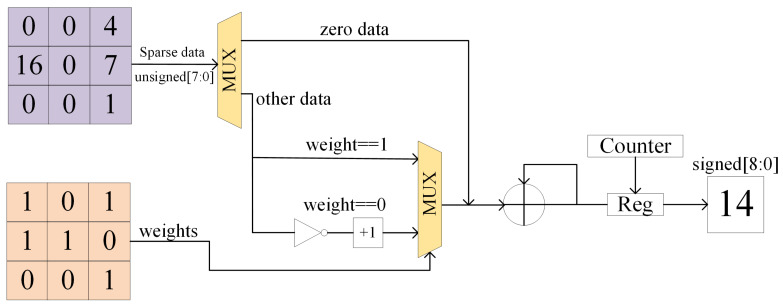
Binary convolution computational processing engine.

The designed sparse binary convolution calculation module is illustrated in Figure 5. MUX is the data selector, Reg is the data register, and Counter is the counter. Because the actual weight value of −1 cannot be represented by 1-bit, we use 0 to replace −1 when storing the weight value. Simultaneously, because the ReLU function processes the feature map data of the previous layer, there are many zero values in the feature map data. During the convolution calculation, the zero values in the feature map will skip the calculation of weights and go directly to the summation stage. The calculation process for the feature map data with numerical values is given by Equation (4), where x denotes the input feature map. After the binary convolution multiplication is completed, the nine data points after the 3×3 convolution are summed eight times into one data point. Data are sent to the accumulation module for further processing.

(4) $$f(x)=\left\{\begin{array}{ll} x & \left(weight=1\right)\\ -x & \left(weight=0\right) \end{array}\right.$$

### 4.4. Multichannel Parallel Compute Design

**Figure 6:**
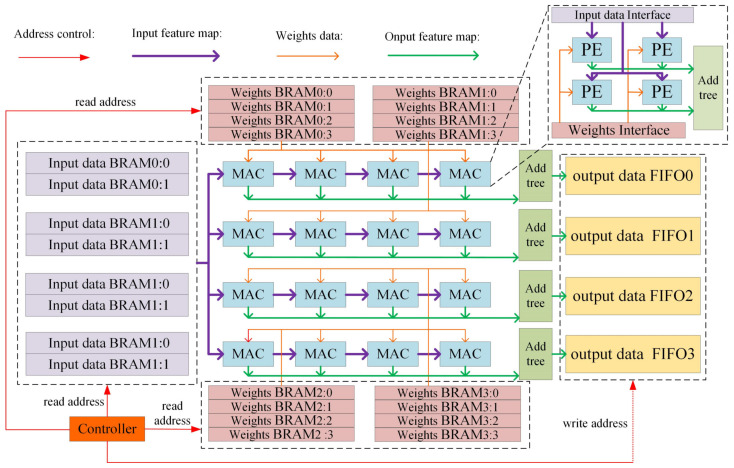
Multichannel Shared Computation architecture.

In the convolutional neural network accelerator architecture design, the data transmission system determines the data transmission bandwidth, and the parallel computing structure determines the data computing speed, which is also a determinant of the CNN acceleration effect. The designed data MCSC architecture is shown in Figure 6, and the storage includes four sets of ping-pong BRAMs that store feature map data and 16 BRAMs that store weights. The computational resources include 16 groups of multiple accumulated (MAC) components. Each set of MAC arrays consists of four binary convolution PEs. In the calculation process, the data read from the BRAM of the input feature map are shared by these 16 groups of MAC arrays, and each group of weight MAC arrays corresponds to a weight storage BRAM. After the feature map and weight data enter the MAC array, multiplication calculations are completed in one clock cycle. Finally, each MAC array outputs the calculation results to an addition tree for the accumulation operation. After the accumulation is completed, it is sent to the output feature map storage FIFO.

**Figure 7:**
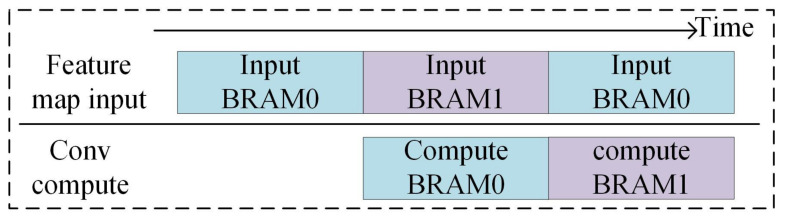
Workflow of ping-pong storage.

Data transfer accounts for a large proportion of the overall system runtime. We designed a pipelined data transfer method based on ping-pong storage to improve computational efficiency. The pipeline transmission architecture is illustrated in Figure 7. The data are first sent to BRAM0 after which, the data are sent to BRAM1, and the data already stored in BRAM0 are calculated simultaneously. When the data in BRAM0 are calculated, the input data are sent to BRAM0, and the data are calculated in BRAM1 when BRAM1 is full. During operation, the idle time of the entire system depends on the difference between the input storage time and the convolution computation time.

### 4.5. Configurable Control Commands

To adapt to the variable-length speech input and forward computation for networks of different sizes, we designed a general instruction set based on AXI. Each instruction had a fixed width of 32 bits. The different instructions include reset, convolution size, number of channels, and storage status. The PS sends the instructions to the PL, and after the instruction processor parses, the controller distributes the relevant instruction information to each module. All modules work together to improve efficiency. The meanings of the customised instructions are listed in Table 4.

**Figure 8:**
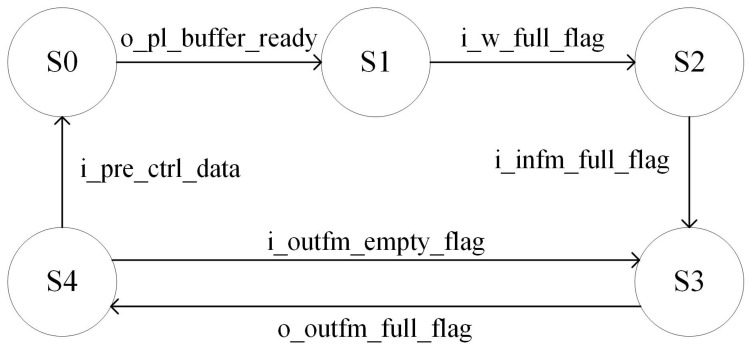
Finite State Machine of convolution compute.

As shown in Figure 8, considering the convolution calculation of the first layer as an example, there are five valid states.

S0 indicates that the storage BRAM is ready to be written, S1 indicates that the weight has been transmitted, S2 indicates that the input feature map data has been transmitted, S3 implies that the convolution calculation has commenced, and S4 indicates that the calculated data are transferred to the DDR. When the first layer of the convolution calculation begins, the process enters state S0. When the storage BRAM at the PL is ready to receive data, it enters the S1 state and starts transmitting the weight data. After the weight data transmission is completed, it enters state S2 to transmit feature map data. After the feature map data are transmitted, they enter state S3 for the convolution calculation. When the FIFO of the output feature map is complete, it enters state S4 and transmits the calculated feature map data back to DDR. If the layer has not been calculated, it returns to state S3 to continue the convolution operation. After all of the calculations are completed, the parameter configuration information of the next layer is sent and returned to state S0.

## 5. Experimental Assessment and Results

In this section, we perform a verification and performance analysis of the entire accelerator. The hardware system was realized based on the MZ7035FA development board of the Xilinx ZYNQ XC7Z035-FFG676-2I chip. The verified model is an improved customised VGG-16 with network parameters of 1W/8A and 8W/8A quantization. This working system design language was Verilog HDL. We synthesized, placed and routed, simulated, and experimented on the accelerator based on the Vivado 2018.2 toolkit. The operating clock frequency was set to 150 MHz.

**Figure 9:**
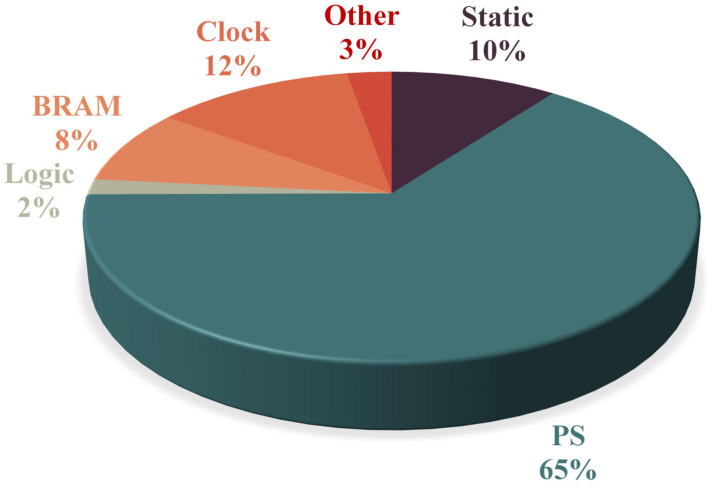
Power consumption breakdown.

The total power consumption was about 2.41 W. Figure 9 depicts the power consumption breakdown. Specifically, PS (containing DDR and DMA) consumed about 65% (1.56 W) of power, Device static consumed about 10% (0.247 W) of power, BRAM consumed nearly 8% (0.203 W) of power, LOGIC consumed about 2% (0.042 W) of power, CLOCK consumed nearly 12% (0.295 W) of power, and other parts consumed 3% (0.066 W) of power. Concretely, the core hardware accelerator logic consumed meagre resources, meeting the needs of low-power Internet-of-Things (IoT) scenarios.

The accelerator system recognised Chinese speech within 8 s. Owing to the configurable system parameters, the processing time for speech of different lengths is variable. As shown in Table 5, for the binary weight network, the content of the 2.24 s speech “stop playing computer” is a continuous daily communication utterance, and the total calculation time is only 69.8 ms. The 8 s speech content “write poems, words, play the piano, violin, and various musical instruments” is a long speech with intervals, and the total calculation time was only 189.5 ms. Compared with the 8-bit weighted network, the processing speed was accelerated by 49.08 ms, and the performance was improved by 20.57%.

To verify the speed improvement on the hardware, the performances of the accelerator and GPU were compared, as shown in Table 6. Among them, the binary neural network had a 2.62 times improvement on the GPU compared with the full-precision network. Compared with the binary network on the GPU, the speed of the binary network after hardware acceleration was improved by 22.08 times.

The performance differences between the proposed accelerator and other BNN accelerators are listed in Table 7. Pipe-CNN [19] is implemented on the ImageNet dataset. Pipe-CNN works in a multiplexed manner, mapping different network layers onto the same hardware unit. Pipe-CNN’s fully pipelined architecture maps all network layers onto slices and transfers different layers to their separate units for optimization. Pipe-CNN achieves excellent throughput, but its power consumption is high, and it is not suitable for edge AI low-power scenarios.

The binary augmented architecture (BAA) [20] is based on the binary augmented pruning (BAP) method that combines binary data and sparsity so that the convolutional layer can reach 98% sparsity. The weights and activation values are encoded. The performance of the BAA in the convolutional layer can reach 31.5 GOP/W. However, the binary convolution unit that we designed skips the sparse activation value directly without additional computation, and the performance in the convolutional layer reaches 35.66 GOPS/W. The work [18] designed a binary neural network accelerator architecture for the 3D convolution design for the behaviour detection dataset KTH, which constrains the weights to 0 and 1 and optimises the convolution structure to improve the data reuse rate. The performance of 14 GOPS/W is achieved on the PYNQ low-resource development board. Our accelerator achieves a performance of 29.36 GOPS/W for the entire model and zero DSP usage in the convolution part for the most extensive network size.

In Table 8, we compare the performance of the accelerator with other hardware designs based on VGG network implementation. For our 1-bit (ours1) and 8-bit (ours2) weighted networks, in terms of hardware, the binary convolutional neural network consumes fewer resources because it does not require a DSP, and fewer registers are required in the convolution calculation process. Furthermore, the binary neural network accelerator takes less time to transmit the same amount of weighted 1-bit data than 8-bit data. Furthermore, the 8-bit network is limited by the number of DSPs and cannot calculate all input data in one clock cycle as in the binary convolution calculation.

For other designs, the works [14,37,38] all used parameterizable configuration and data multiplexing, in which the authors of [14] optimised the instructions, used the ping-pong storage method, and [38] reduced the number of data accesses through kernel partitioning. The works of [38,39] implemented hardware using advanced chip technology on Intel FPGAs. The authors of [39] constructed a generic CNN compiler to generate customised FPGA hardware for different CNN inference tasks. The authors of [40] designed a Bayesian convolution-based method to improve the uncertainty and regularisation of traditional CNNs for 2D and 3D vision tasks, balancing the requirements between algorithms and hardware. The authors of [41] propose a sparse-wise dataflow to skip the cycles of processing multiply-and-accumulates (MACs) with zero weights and exploit data statistics to minimize energy through zeros gating to avoid unnecessary computations. Our work algorithmically uses a binary weighted convolution method to build neural networks with a low accuracy loss. In terms of hardware, a parameterizable configuration, data multiplexing, and ping-pong storage were realised. Moreover, the MCSC architecture was established such that the convolution multiplication can be calculated in one cycle and does not require DSP resources. The proposed binary neural network accelerator demonstrated the lowest power consumption. Simultaneously, the usage of the accelerator LUT was significantly reduced because the binary convolution computation uses fewer register resources. The accelerator performance at the convolutional layer was 35.66 GOPS/W, which was 1.11 times of [41] and 1.29 times of [38]. The overall neural network accelerator performance was 29.36 GOP/W, which was 2.07 times of [37], 1.37 times of [14], 1.21 times of [39] and 1.5 times of [40]. The performance of ours1 with different weights and bit widths under the same design improved by 1.32 times, which verifies the effectiveness of the designed binary neural network accelerator.

**Table 8:** Comparison with other implementations for VGG networks.

	TCAD ’17 [37]	TCAD ’18 [39]	TVLSI ’20 [14]	TVLSI ’20 [41]	TCAS-II ’21 [38]	TCAD ’22 [40]	Ours1	Ours2
Platform	ZYNQ XC7045	Intel Arria 10GX 1150	Kintex-7XC7 K325T	ZYNQ ZCU102	Virtex-7VC709	Intel Arria 10GX1150	ZYNQ 7035	ZYNQ 7035
Technology	28 nm	20 nm	28 nm	20 nm	28 nm	20 nm	28 nm	28 nm
Network	VGG-16	VGG-16	VGG-16	VGG-16	VGG-16	Bayes VGG-11	Customized VGG-16	Customized VGG-16
Clock (MHz)	150	240	200	200	200	220	150	150
Precision	16W/16A	8W/16A	8W/8A	16W/16A	8W/8A	8W/8A	8W/8A	1W/8A
LUT	182,616	208,000	94,763	390,000	121,472	427,200	79,497	63,968
FF	127,653	-	150,848	-	159,872	1,708,800	74,498	51,998
BRAM	486	2319	165	1460	467	2731	408	374
DSP	780	3036	516	1352	664	1518	144	0(C) 64(O)
GOPS	137	968.03	354	495.4	230.1	533.75	64.5(C)	85.95(C)
							56.1(O)	70.77(O)
Power (W)	9.63	40	16.5	15.4	9.61	43.6	2.528	2.41
GOPS/W	14.2(O)	24.3(O)	21.45(O)	32.16(C)	23.9(C)	19.6(O)	25.5(C)	35.66(C)
							22.19(O)	29.36(O)

Note: C = conv. O = overall. W = weight fixed-point bit width. A = activation fixed-point bit width.

## 6. Conclusions

In this study, an improved binary weight VGG network model was developed based on the robustness of the convolutional neural network, which compresses the parameters of the convolutional layer by more than 27 times. We designed a general neural network accelerator whose parameters can be configured with instructions based on the characteristics of variable-length speech input. Then, based on the 32-bit data transmission bit width of the AXI bus, the convolution four-matrix fetching method and binary convolution PE without DSP were designed. Furthermore, we proposed an MCSC architecture that shares data, computes arrays, and uses ping-pong BRAM for input data storage. To ensure that the accuracy is not quantised during the convolution calculation, only the feature map is dynamically quantised using the activation function. Our experiments on ZYNQ 7035 consumed only 2.41 W at a work clock frequency of 150 MHz, and the convolution part achieved a performance of 35.66 GOPS/W. The software–hardware co-design approach makes AI applications more efficient. We aim to explore the possibility of binarizing fully-connected layers with guaranteed accuracy in future studies to achieve more lightweight models and enhanced computing power.

## Figures and Tables

**Table 1:** Customize VGG-16 Speech Recognition Network Topology Analysis.

Layers	Topology ^a^	Kernel Size	Output Shape	FP ^b^ Parm	BW ^c^ Parm	MACS
Input	-	-	800×64×1	-	-	-
Layer-1	Conv + ReLU	3×3×1×32	800×64×32	1.25K	0.16K	29,491,200
Layer-2	Conv + ReLU + MP	3×3×32×32	400×32×32	36.125K	1.375K	943,718,400
Layer-3	Conv + ReLU	3×3×32×64	400×32×64	72.25K	2.5K	471,859,200
Layer-4	Conv + ReLU + MP	3×3×64×64	200×16×64	144.25K	4.75K	943,718,400
Layer-5	Conv + ReLU	3×3×64×128	200×16×128	288.5K	9.5k	471,859,200
Layer-6	Conv + ReLU	3×3×128×128	200×16×128	577k	19K	943,718,400
Layer-7	Conv + ReLU + MP	3×3×128×128	100×8×128	577K	19k	943,718,400
Layer-8	Conv + ReLU	3×3×128×256	100×8×256	1154K	38k	471,859,200
Layer9	Conv + ReLU	3×3×256×256	100×8×256	2308K	76K	943,718,400
Layer10	Conv + ReLU	3×3×256×256	100×8×256	2308K	76K	943,718,400
Layer11	Conv + ReLU	3×3×256×256	100×8×256	2308K	76K	943,718,400
Layer12	Conv + ReLU	3×3×256×256	100×8×256	2308K	76K	943,718,400
Layer13	Conv + ReLU	3×3×256×256	100×8×256	2308K	76K	943,718,400
Layer14	Conv + ReLU	3×3×256×256	100×8×256	2308K	76K	943,718,400
Layer15	Conv + ReLU	3×3×256×256	100×8×256	2308K	76K	943,718,400
Layer16	Conv + ReLU	3×3×256×256	100×8×256	2308K	76K	943,718,400
Layer17	FC + Softmax	2048×1424	100×1424	11392K	-	583,270,400
total	-	-	-	18.71M(C) 29.83M(O)	0.68M(C) 11.81M(O)	13.35296G

^a^ MP = MaxPooling, FC = fully-connected layer. ^b^ FP = full precision. ^c^ BW = binary weight. Note: C = Conv and O = Overall.

**Table 2:** Accuracy of customized VGG-16 and other networks on ST CMD.

Networks	Conv Parameters	Data Format	PER
RRAINet [23]	-	Float 32-bit	15.45%
MCNN [24]	-	Float 32-bit	22.97%
VGG-CTC [26]	-	Float 32-bit	23.86%
Customized VGG-16	18.71M	Float 32-bit	15.86%
Customized VGG-16	9.36M	16 W/16A	16.25%
Customized VGG-16	4.68M	8W/8A	16.37%
Customized VGG-16	0.68M	1W/Float 32-bit	16.13%
Customized VGG-16	0.68M	1W/16A	17.90%
Customized VGG-16	0.68M	1W/8A	17.91%

Note: W = weight fixed-point bit width, A = activation fixed-point bit width.

**Table 3:** Comparison of different data transmission systems.

Data Transmission Method	Hardware Resource Cost	Maximum Transfer Speed
AXI_HP DMA	high	390 m/s
AXI_HP CDMA	medium	194 m/s
AXI_ACP CDMA	medium	144 m/s

**Table 4:** Configurable Control Instruction Interpretation.

Direction	Instruction	Function
PS to PL	i_rst_n	Accelerator overall reset
	i_pre_ctrl_data	Clear storage and padding flag, network width, height and channels
	i_infm_full_flag	Input feature map BRAM full flag
	i_w_full_flag	Weight BRAM full flag
	i_outfm_empty_flag	Output feature map FIFO empty flag
PL to PS	o_pl_buffer_ready	BRAM is ready to receive the data flag
	o_outfm_full_flag	Output feature map FIFO full flag
	o_infm_empty_flag	Input feature map BRAM empty flag
	o_w_empty_flag	Weights BRAM Empty Flag
	o_wdense_full_flag	Full connection layer weight full flag
	o_fbank_number	The length of this speech

**Table 5:** The computational time of each layer for different speech.

Layer	2.24 s Speech Calculation Time (ms)		8 s Speech Calculation Time (ms)	
	8W/8A	1W/8A	8W/8A	1W/8A
Conv1	0.81	0.88	2.85	2.46
Conv2 + MaxPool	5.20	2.75	16.84	7.61
Conv3	2.10	1.47	6.62	4.52
Conv4 + MaxPool	5.35	2.66	17.20	7.57
Conv5	2.23	1.61	6.43	4.84
Conv6	4.11	2.91	12.23	9.13
Conv7 + MaxPool	4.04	2.71	11.95	8.66
Conv8	3.34	2.25	7.43	6.11
Conv9-16	51.82	34.11	116.36	97.93
Dense + Softmax	18.45		40.68	
total	97.45	69.80	238.59	189.51

**Table 6:** Comparison with GPU.

	GeForce RTX TITAN		ZYNQ 7035	
Frequency (MHz)	837		150	
Speech length (s)	8		8	
Precision	32W/32A	1W/32A	8W/8A	1W/1A
Power (W)	250		2.52	2.41
Speedup	1.0×	2.62×	41.62×	52.4×
Time (ms)	9930.78	3790.37	238.59	189.51

**Table 7:** Comparison with other implementations for binary neural networks.

	FCCM ’20 [19]	T-NNLS ’21 [20]	JRTIP ’22 [18]	Ours
Platform	VirtexUSXCVU9P	ZYNQ ZC706	PYNQ-Z2	ZYNQ 7035
Technology	28 nm	28 nm	28 nm	28 nm
Network	Pipe-CNN ImageNet	AlexNet	3D AND Net	Customized VGG-16
MACs	0.621G	2.27G	58.33M	13.35G
Clock (MHz)	300	166	100	150
Precision	1W/8A	1W/8A	1W/1A	1W/8A
LUT	27,4795	218,000	21,727	63,968
BRAM	2746	545	140	374
DSP	2370	900	14	0(C) 64(O)
GOPS	2419(C)	71.91(O) 292.97(C)	22.4	85.95(C)
				70.77(O)
Power(W)	75	9.3	1.6	2.41
GOPS/W	32.3(C)	31.5(C) 7.73(O)	14(O)	35.66(C)
				29.36(O)

Note: C = conv. O = overall. W = weight fixed-point bit width. A = activation fixed-point bit width.

## Data Availability

The speech recognition data set ST SMD used in this paper comes from https://www.surfing.ai (accessed on 14 December 2022).
